# Comparison of Outpatient Health Care Use Before and After Pediatric Severe Sepsis

**DOI:** 10.1001/jamanetworkopen.2020.15214

**Published:** 2020-09-10

**Authors:** Erin F. Carlton, Joseph G. Kohne, Matthew K. Hensley, Hallie C. Prescott

**Affiliations:** 1 Division of Critical Care Medicine, Department of Pediatrics, University of Michigan, Ann Arbor; 2 Susan B. Meister Child Health Evaluation and Research Center, Department of Pediatrics, University of Michigan, Ann Arbor; 3 Division of Pulmonary, Allergy and Critical Care Medicine, University of Pittsburgh, Pittsburgh, Pennsylvania; 4 Department of Internal Medicine, Division of Pulmonary and Critical Care Medicine, University of Michigan, Ann Arbor; 5 US Department of Veterans Affairs Center for Clinical Management Research, Health Services Research and Development Center of Innovation, Ann Arbor, Michigan

## Abstract

This cohort study examined the number and type of outpatient health visits before and after hospitalization for pediatric severe sepsis.

## Introduction

Severe sepsis hospitalizes more than 70 000 children each year in the United States, with costs exceeding $7.3 billion.^1^ More than 90% of children who experience sepsis hospitalization survive. However, 1 in 6 children is rehospitalized within 30 days, a rate markedly higher than that for other common pediatric hospitalizations.^2^ We hypothesized that outpatient health care is similarly greater following pediatric sepsis hospitalization than before this hospitalization. To test this hypothesis, we measured the change in outpatient health care visits among patients who survived pediatric sepsis.

## Methods

This cohort study followed the Strengthening the Reporting of Observational Studies in Epidemiology (STROBE) reporting guideline. The University of Michigan determined that the study did not require institutional review board review and was exempt from informed consent because the database used is deidentified. We studied pediatric severe sepsis hospitalizations in Optum’s deidentified Clinformatics Data Mart database (2010-2015), which contains health care claims from commercial and Medicare Advantage members. Neonatal and pregnancy-related hospitalizations were excluded. We identified severe sepsis by either (1) an explicit diagnosis code (using *International Classification of Diseases, Ninth Revision [ICD-9]* and *International Statistical Classification of Diseases and Related Health Problems, Tenth Revision [ICD-10]* coding systems) for severe sepsis or septic shock or (2) concurrent codes for sepsis or bacteremia and acute organ dysfunction.^3^ Comorbidities were measured using the Complex Chronic Conditions Classification algorithm.^4^

We compared the number of outpatient visits in the year before vs the year after a severe sepsis hospitalization using a *t* test, and we compared the type of outpatient visits in the year before and the year after a severe sepsis hospitalization using a χ^2^ test. Outpatient visits included all primary care and subspecialty visits. We determined the percentage of patients with new subspecialty visits as the proportion with a subspecialty visit in the 90 days after sepsis hospitalization and no visit to that subspecialty in the preceding year. In our primary analysis, we required that children be consecutively enrolled in insurance for 3 or more months prior to sepsis hospitalization, but we performed several sensitivity analyses (eAppendix in the Supplement).

Analyses were performed from January 2020 to April 2020 using Stata/MP statistical software version 15 (StataCorp). *P* values were 2-sided, and statistical significance was set at *P* < .05.

## Results

Of 167 497 pediatric hospitalizations, 952 were for severe sepsis (0.6%); of patients hospitalized for severe sepsis, 855 (89.8%) survived to discharge and were included in our analysis (Table). This cohort had a median (interquartile range [IQR]) age of 12 (3-16) years, with median (IQR) length of stay of 9 (5-20) days; 404 patients (47.3%) were girls.

**Table.** Cohort Characteristics and Outpatient Health Care Use

Characteristic	Individuals, No. (%)		
	All (n = 855)	Without comorbidity (n = 493)	With ≥1 comorbidities (n = 362)
Age, median (IQR), y	12 (3-16)	13 (4-17)	9 (2-15)
Girls	404 (47.3)	238 (48.3)	166 (45.8)
Hospital length of stay, median (IQR), d	9 (5-20)	7 (4-14)	12.5 (5-27)
Outpatient health care use			
Visits in 90 d, median (IQR)			
Before sepsis hospitalization	2 (1-4)	1 (0-3)	3 (1-6)
After sepsis hospitalization	3 (1-6)	3 (1-5)	4 (2-7)
Total visits in 365 d, median (IQR)			
Before sepsis hospitalization	5 (2-11)	3 (1-8)	8 (3-17)
After sepsis hospitalization	8 (3-15)	6 (3-12)	12 (6-20)
New subspecialty visits, No./total No. without prior subspecialty visit^a^			
Any subspecialist	137/855 (16.0)	60/493 (12.2)	77/362 (21.3)
≥2 Subspecialists	22/855 (2.6)	11/493 (2.2)	11/362 (3.0)
Hematology/oncology	31/722 (4.3)	10/457 (2.2)	21/265 (7.9)
Pulmonology	33/802 (4.1)	17/475 (3.6)	16/327 (4.9)
Nephrology	29/833 (3.5)	15/487 (3.1)	14/346 (4.0)
Gastroenterology	27/752 (3.6)	16/450 (3.6)	11/302 (3.6)
Cardiology	26/799 (3.3)	10/472 (2.1)	16/327 (4.9)
Neurology	20/788 (2.5)	8/473 (1.7)	12/315 (3.8)

Abbreviation: IQR, interquartile range.

^a^ Outpatient visits were identified by a type of service variable specific to outpatient professional services and included all outpatient clinician encounters. The percentage of new subspecialists was determined by the number of patients with a new subspecialty visit within 90 days of discharge among those patients who did not have a subspecialty visit in the 365 days prior to sepsis hospitalization. New subspecialty designation was based on an individual subspecialty (eg, a patient did not have a pulmonology appointment prior to sepsis hospitalization but did after hospitalization).

Outpatient visits increased from a median (IQR) of 5 (2-11) visits per patient in the year prior to hospitalization for severe sepsis to 8 (3-15) visits in the year after hospitalization (*P* < .001) and from 2 (1-4) visits in the 90 days before hospitalization for severe sepsis to 3 (1-6) visits in the 90 days after hospitalization (*P* < .001) (Figure). Of 855 patients, 478 (55.9%) had more visits in the 90 days after hospitalization vs before hospitalization and 514 (60.1%) had more visits in the year after sepsis hospitalization vs the year before hospitalization. Similar to the primary analysis, the median number of outpatient visits increased in the year after sepsis in all sensitivity analyses.

**Figure.**
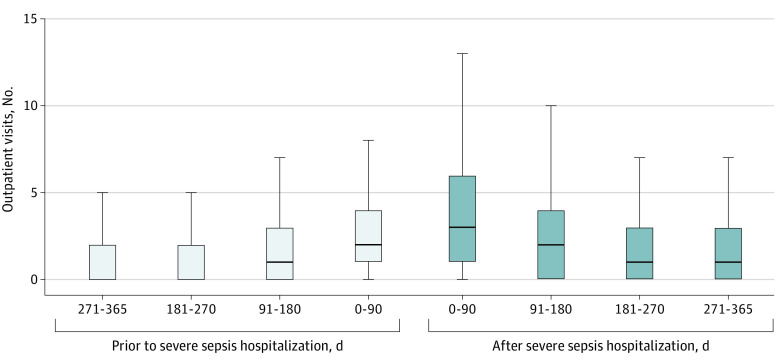
Outpatient Health Care Use by Quarter in the Year Before and After Severe Sepsis Middle lines indicate medians; boxes, interquartile ranges; whiskers, the upper and lower adjacent values.

Of 855 patients, 137 (16.0%) had a new subspecialist visit in the 90 days after sepsis hospitalization, most often for hematology and oncology (31 patients [4.3%]) or pulmonology (33 patients [4.1%]). Of 493 previously healthy children, 285 (57.8%) had an increase in outpatient visits by 90 days after sepsis hospitalization, while of 362 children with complex chronic conditions, 193 (53.3%) had an increase in such visits (*P* = .19). Among 493 previously healthy children, 60 (12.2%) had a new subspecialist visit, while of 362 children with complex chronic conditions, 77 (21.3%) had a new subspecialist visit (*P* < .001).

## Discussion

In this national cohort of pediatric sepsis survivors, the median number of outpatient visits following sepsis hospitalization increased by 60% in the year after hospitalization for sepsis compared with each patient’s own baseline. Nearly 1 in 6 children had a new subspecialist visit within 3 months of severe sepsis hospitalization. However, not all children had an increase in outpatient visits, highlighting the heterogeneity of postsepsis experiences among children.

The study has some limitations. This cohort consisted of privately insured children, so the results may not extrapolate to children on Medicaid or other types of insurance. We were unable to measure referrals or the number of patients who missed scheduled appointments. We used a claims-based algorithm to identify hospitalizations for severe sepsis, and that method may have resulted in some misclassification.^5^ This cohort study found that severe sepsis hospitalization was associated with an increase in subsequent outpatient primary care and subspecialty visits for most children who experience such hospitalization. That association may have a significant impact on children and their families, including potentially contributing to a financial burden on families, a decrease in parental work, and increased school absenteeism for children.

## Notes

Supplement. **eAppendix.** Supplementary Methods
