# Supplementary material for: Comparison of Outpatient Health Care Use Before and After Pediatric Severe Sepsis
Source: JAMA Netw Open. 2020 Sep 10;3(9):e2015214. doi: 10.1001/jamanetworkopen.2020.15214 (PMC7489846; doi:10.1001/jamanetworkopen.2020.15214)
Supplement: Supplement. — eAppendix. Supplementary Methods [file jamanetwopen-e2015214-s001.pdf]

Supplementary Online Content

Carlton EF, Kohne JG, Hensley MK, Prescott HC. Comparison of outpatient health care use before and after pediatric severe sepsis. *JAMA Netw Open*. 2020;3(9):e2015214. doi:10.1001/jamanetworkopen.2020.15214

eAppendix. Supplementary Methods

This supplementary material has been provided by the authors to give readers additional information about their work.

© 2020 Carlton EF et al. *JAMA Network Open.*

eAppendix. Supplementary Methods

Sensitivity Analysis: In our primary analysis, all patients were required to have insurance coverage for at least 3 months prior to index sepsis hospitalization. However, to assess whether lack of consistent insurance coverage either before or after sepsis hospitalization may have biased our results, we completed four sensitivity analyses. First, we excluded patients under 1 year of age during sepsis hospitalization (to allow for a full year of data prior to sepsis hospitalization). Second, we examined only those patients who had consecutive insurance for 6 months prior to and following sepsis hospitalizations, as inferred by presence of claims at least 6 months before and after sepsis hospitalization. We similarly measured outpatient healthcare visits among patients with consecutive insurance coverage for the 9 months and 12 months preceding and following sepsis hospitalization, again as inferred by presence of claims before and after these time periods. These four sensitivity analyses show a similar increase in outpatient healthcare use with varying length of enrollment and analysis period.

© 2020 Carlton EF et al. *JAMA Network Open.*
